# Electrospun poly(caprolactone)-elastin scaffolds for peripheral nerve regeneration

**DOI:** 10.1007/s40204-014-0020-0

**Published:** 2014-02-21

**Authors:** Katelyn E. Swindle-Reilly, Chinmay S. Paranjape, Cheryl A. Miller

**Affiliations:** 1 Department of Biomedical Engineering, Saint Louis University, 3507 Lindell Blvd., St. Louis, MO 63103 USA; 2 Department of Chemical and Biomolecular Engineering, University of Pennsylvania, 220 South 33rd Street, Philadelphia, PA 19104-6315 USA

**Keywords:** Electrospinning, Elastin, PCL, Peripheral nerve regeneration, Dorsal root ganglia, Nanofibers

## Abstract

Peripheral nerve regeneration can be enhanced by chemical and mechanical cues for neurite growth. Aligned and randomly oriented electrospun nanofibers of poly(ε-caprolactone) (PCL) or a blend of PCL and elastin were fabricated to test their potential to provide contact guidance to embryonic chick dorsal root ganglia for peripheral nerve regeneration. Scanning electron microscopy was used to analyze the fiber diameter. Fiber diameter was found to be significantly smaller when elastin was incorporated into the scaffold (934 ± 58 nm for PCL and 519 ± 36 nm for PCL:elastin). After 24 h in culture, there was preferential cell attachment and neurite extension along the fibers of the elastin-containing scaffolds (average neurite extension 173.4 ± 20.7 μm), indicating that the presence of elastin promotes neurite outgrowth on electrospun scaffolds.

## Introduction

Hundreds of thousands of cases of peripheral nerve injury occur each year in the United States and Europe. These injuries result in loss of motor function, sensation, or neuropathic pain (Griffin and Hoffman 1993). Peripheral nerves require guidance mechanisms to regenerate over gaps larger than 1 cm. A tissue engineering scaffold implanted to bridge the gap between nerve ends can promote guided regeneration. Electrospun degradable polymer scaffolds are ideal due to controllable fiber size, degradability, and incorporation of stimulants. Nanofibers have approximately the same diameter as the native fibrous extracellular matrix (ECM) proteins (Yu et al. 2008). In addition, it has been shown that cells adhere to and align along the length of nanofibers. Differentiation rate of neural stem cells has been shown to be higher on nanofibers than microfibers (Yang et al. 2005).

Electrospinning can be used to control thickness, composition, and porosity of tissue engineering scaffolds. Electrospinning can be used to produce nanofibers using an electric field to draw the polymer from an orifice to a grounded collector. Mutual charge repulsion by the polymer helps the polymer overcome Taylor cone surface tension to form a jet. As the solvent dries, nanofibers collect on the plate (Venugopal et al. 2008). Electrospun nanofibers have a large surface area and porosity that favor cell adhesion and spreading. High fiber porosity enables nutrient exchange for cultured cells (Panseri et al. 2008). These fibers can be collected in random orientation or aligned. Alignment has been shown to influence the direction of cell spreading, especially for neural cells (Chew et al. 2008; Corey et al. 2007).

The ideal nerve guide should not swell or change shape, should degrade slowly, and must be capable of bearing mechanical stress during the surgical implantation. Electrospun fibers have these characteristics. The most commonly used polymers for electrospinning for tissue engineering applications are poly(l-lactic acid) (PLLA) (Corey et al. 2007; Kim et al. 2008; Koh et al. 2008; Panseri et al. 2008; Yang et al. 2005), poly(lactide-co-glycolide) (PLGA) (Li et al. 2006a, b; Panseri et al. 2008; Stitzel et al. 2006), and poly(ε-caprolactone) (PCL) (Chew et al. 2008; Ghasemi-Mobarakeh et al. 2008; Panseri et al. 2008; Reed et al. 2009; Schnell et al. 2007; Zhang et al. 2005). These polymers can be used alone or with incorporated elements to overcome the hydrophobicity of the fibers.

The addition of natural polymers can improve cell adhesion. Investigators have used a combination of gelatin (Ghasemi-Mobarakeh et al. 2008; Kim et al. 2008; Li et al. 2005, 2006a; Powell and Boyce 2008; Zhang et al. 2005), laminin (Koh et al. 2008; Neal et al. 2009, 2012; Wen and Tresco 2006), fibronectin (Wen and Tresco 2006), collagen (Boland et al. 2004; Buttafoco et al. 2006; Corey et al. 2007; Li et al. 2005; Matthews et al. 2002; Schnell et al. 2007; Stitzel et al. 2006), chitosan (Desai et al. 2008), hyaluronic acid (Um et al. 2004), and elastin (Boland et al. 2004; Buttafoco et al. 2006; Li et al. 2005, 2006a; Stitzel et al. 2006) to enhance biocompatibility and cell proliferation. Biocomposites are desirable due to the fibrous nature of the synthetic backbone that imparts mechanical strength while the ECM components act as promoters for cell attachment and growth (Ghasemi-Mobarakeh et al. 2008). The ECM molecules shape progenitor cell migration and differentiation in addition to guidance of new axons (Yu et al. 2008).

Recent research has shown promising results using electrospun scaffolds containing biopolymers blended with synthetic polymers for nerve regeneration (Ghasemi-Mobarakeh et al. 2008; Sell et al. 2010). Neal et al. (2009, 2012) investigated the incorporation of laminin, a basement membrane protein known to enhance nerve regeneration. Prabhakaran et al. (2013) evaluated a combination of PHBV with collagen, another basement membrane protein, for nanofiber substrates for nerve tissue engineering. In addition, alignment of nanofibers has been shown to be preferential for nerve regeneration (Jha et al. 2011), and micropatterning further enhances nerve regeneration (Jeffries and Wang 2012).

Other researchers have developed nanofiber scaffolds based on PCL, blends of collagen and elastin (Li et al. 2005), or blends of PLGA and elastin (Li et al. 2006a), but none have reported the combination of PCL and elastin for application in peripheral nerve regeneration. We have selected a copolymer of various blends of PCL and elastin dissolved in hexafluoro-2-propanol (HFP) to produce electrospun scaffolds. PCL provides a controlled degradation rate under physiological conditions while elastin promotes cell attachment and scaffold elasticity (Li et al. 2005). Microscopy revealed repeatable nanofiber scaffolds were produced using electrospinning parameters of 15 kV and 16 cm collection distance. Day 9 embryonic chick dorsal root ganglia (E9 DRG) were seeded on the electrospun scaffolds for tissue culture studies, and scanning electron microscopy (SEM) was used to determine the fiber diameter.

## Methods

### Scaffold fabrication

Poly(ε-caprolactone) (PCL) and elastin derived from bovine neck ligament were dissolved in hexafluoro-2-propanol (HFP) (Sigma-Aldrich) at 15 % w/v and 20 % w/v, respectively. Two solutions were used in the electrospinning studies: 15 % PCL or a 4:1 blend of PCL:elastin. The final concentrations of PCL and elastin in the blend were 12 and 4 %, respectively. A standard electrospinning apparatus was used in which a high-voltage power supply (Spellman CZE 1000R) was used to hold the voltage constant at 15 kV and the tip to collection plate distance was 16 cm. Briefly, the polymer solution was delivered through tubing to a charged 18G needle tip at a controlled flow rate of 30 μL/min (Cole-Parmer MasterFlex C/L 77120-52). For random configuration, the fibers were collected on an aluminum plate, and for aligned fibers, the collection occurred between two bolts attached to the collection plate (Wang et al. 2009).

### Scaffold structure evaluation

The average fiber diameter of electrospun scaffolds was examined via scanning electron microscopy (SEM) (JEOL JSM 5800) using techniques from Powell and Boyce (2008). Desiccated PCL scaffolds were mounted onto 12 mm aluminum stubs, sputter coated with gold–palladium, and imaged at 15 kV accelerating voltage. Fiber diameters were determined via image analysis using ImageJ. The porosity of dry scaffolds was determined by weighing PCL meshes and then finding their volume via water displacement in a 100 μL syringe in vacuum. The porosity (free volume) was then calculated by comparing the density of the electrospun meshes to that of a solid PCL disk of the same volume (Powell and Boyce 2008). Porosity was calculated using the method from Zhu et al. (2008).

### Cell culture

The scaffolds were desiccated, washed with ethanol, and sterilized under UV light for 1 h prior to cell culture experiments. Day 9 embryonic chick dorsal root ganglia (E9 DRG) were extracted, dissociated, and seeded at a cell density of 2 × 10^4^ cells/mL on the electrospun scaffolds in F12k media supplemented with 20 % fetal bovine serum (FBS) and 25 ng/mL nerve growth factor (NGF). The cultures were incubated at 37 °C in 5 % CO_2_ for 24 h.

### Morphological evaluation

Immunohistochemistry was performed in order to assess cell adhesion and neurite extension. Cell adhesion was observed by fixing the cells with 10 % neutral buffered formalin and labeling with Hoechst 33258. Neurite extension was determined by primary antibody (beta-III-tubulin) application for 1 h, three 1 min washes in PBS, and then application of the secondary antibody (AlexaFluor 488) for 30 min. Samples were imaged using fluorescent microscopy on an inverted Zeiss microscope equipped with X-Cite 120 Fluorescence Illumination System. Neurite measurements were obtained using AxioVision software.

### Statistical analysis

All results shown are expressed as mean ± standard error of mean (SE). A student *t* test was used to determine statistical significance at *p* < 0.05.

## Results and discussion

**Fig. 1:**
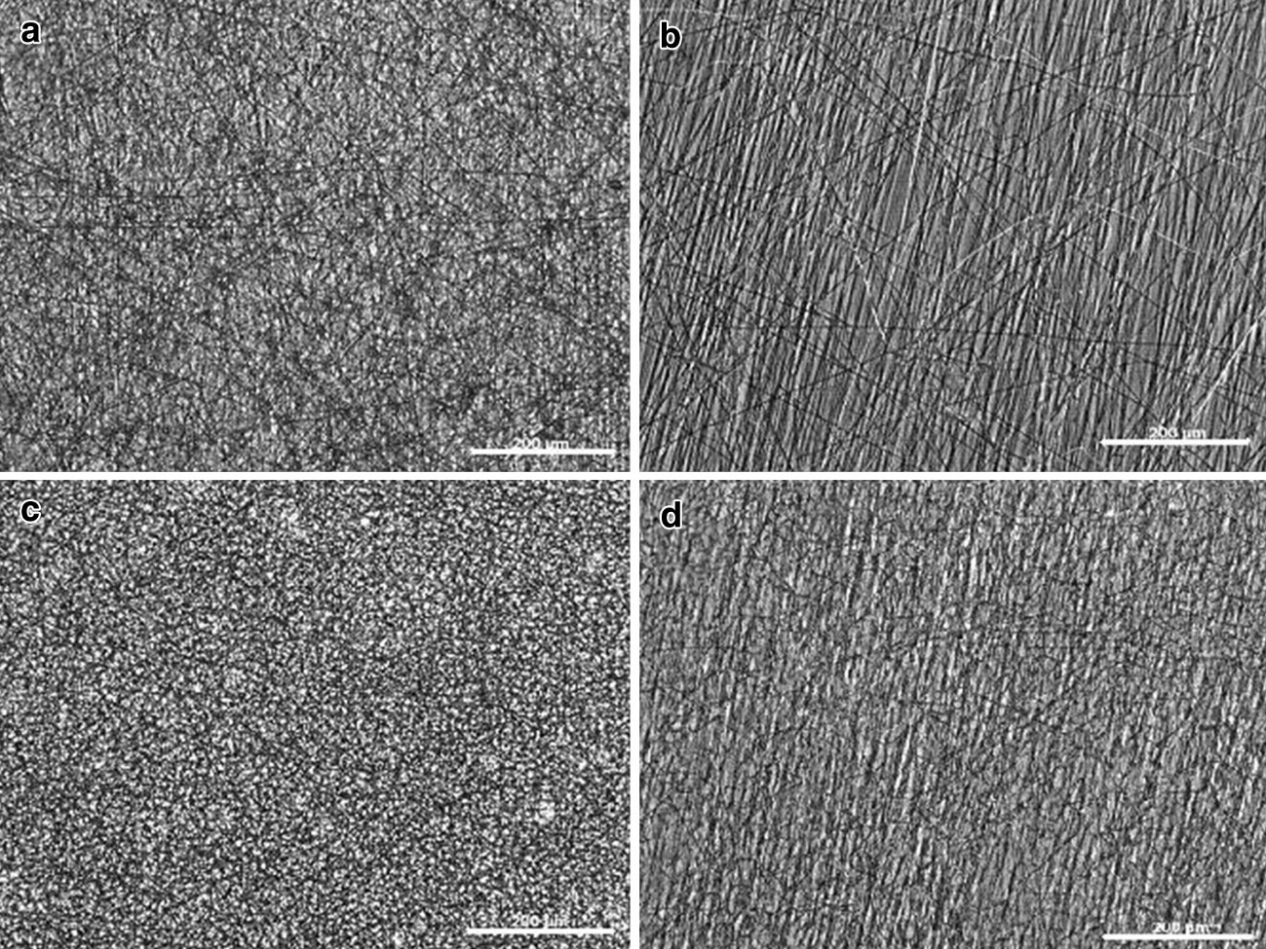
Light microscopy images of **a** random PCL, **b** aligned PCL, **c** random PCL:elastin, **d** aligned PCL:elastin. *Scale bar* is 200 μm

Electrospinning was used to produce nanofiber scaffolds. It was found that increasing the voltage to 15 kV and the collection distance to 16 cm produced nanofibers with minimal bead defects. In particular, the higher voltage was required to produce scaffolds containing elastin without bead defects. Light microscopy images of the PCL and PCL:elastin scaffolds are shown in Fig. 1.

SEM was used to image the individual fibers and to determine the fiber diameter. The fiber diameters (*n* = 9) are reported in Table 1. The PCL and PCL:elastin fiber diameters were statistically different at 934 and 519 nm, respectively. The differences between the fiber diameters for the aligned and random orientations were not significantly different. Incorporation of elastin into the nanofiber network, therefore, significantly decreased the fiber diameter with all other conditions held constant.

**Table 1:** Average fiber diameter ± standard error (*n* = 9)

Composition	Alignment	Fiber diameter (nm)
PCL	Random	934 ± 58
PCL	Aligned	1,030 ± 84
PCL:elastin	Random	519 ± 36
PCL:elastin	Aligned	416 ± 29

**Fig. 2:**
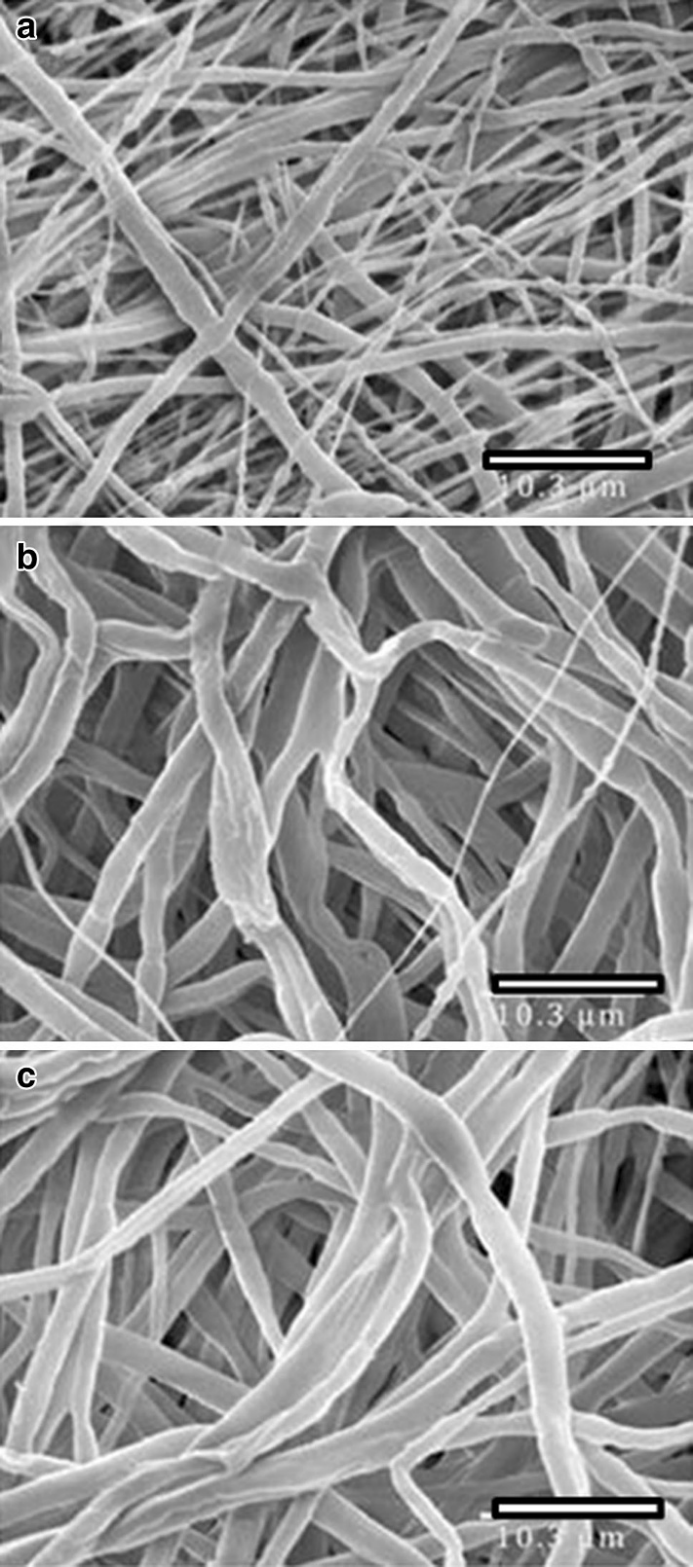
SEM micrographs of PCL nanofibers produced from **a** 15 % solution, **b** 20 % solution, **c** 25 % solution. *Scale bar* is 10.3 μm

Polymer concentration was varied to optimized fiber diameter and scaffold porosity. SEM micrographs in Fig. 2 show increased fiber diameter for higher concentration polymer solution. Higher polymer concentration also resulted in decreased porosity. Therefore, 15 % PCL polymer solution was used for all studies (porosity 63.3–80.5 %). Fiber morphology varied due to polymer composition. SEM showed round, uniform fibers when PCL alone was used in electrospinning. The incorporation of elastin into the polymer blend decreased the fiber diameter and caused minimal bead defects. Higher magnification SEM images (not shown) also reveal that incorporation of elastin into the scaffolds produces fibers with a flatter, more ribbon-like appearance compared to the tubular fibers obtained using just PCL. Incorporation of elastin has been previously shown to result in ribbon-like fibers (McClure et al. 2008).

**Fig. 3:**
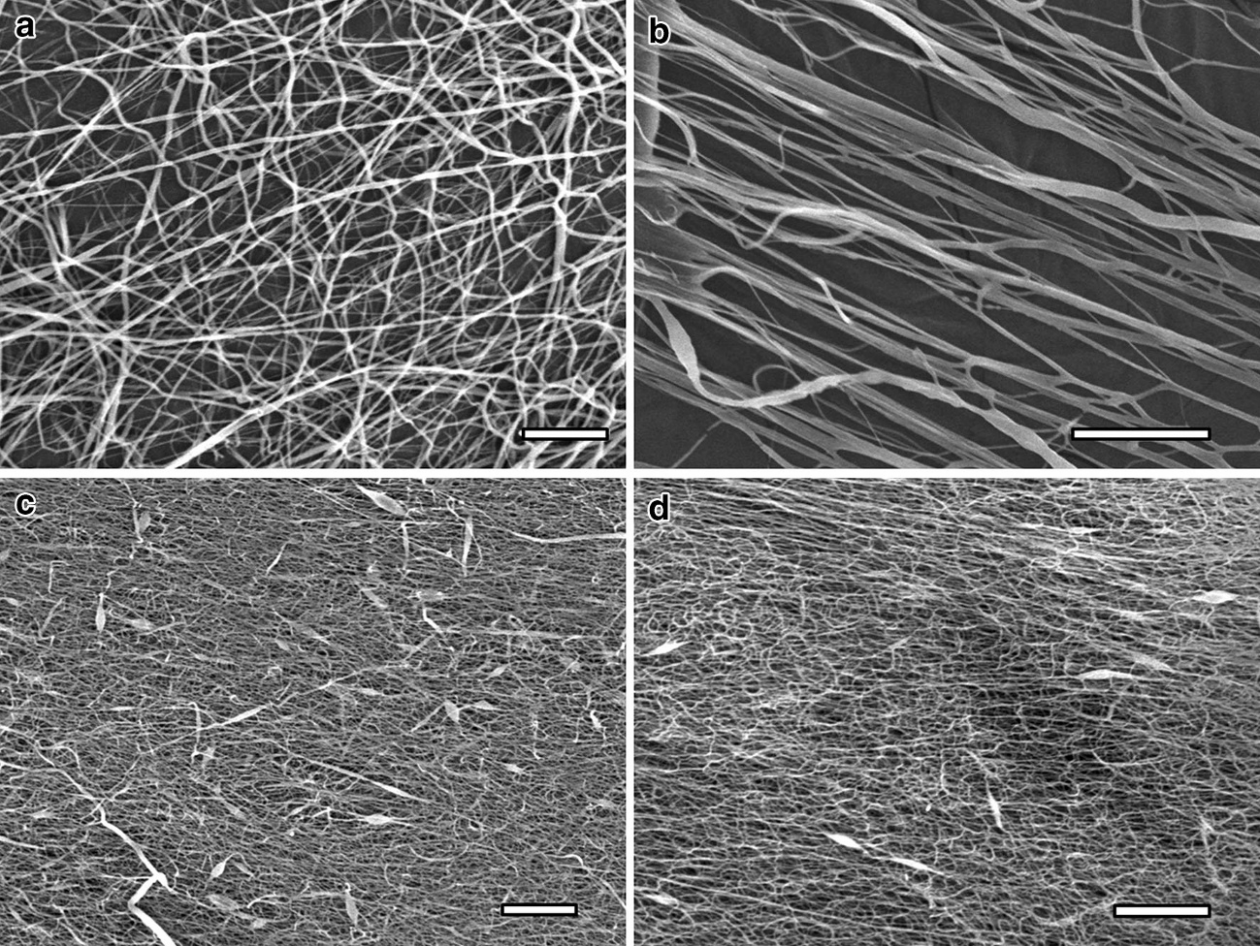
SEM micrographs of **a** random PCL fibers, **b** aligned PCL fibers, **c** random PCL:elastin fibers, **d** aligned PCL:elastin fibers. *Scale bar* is 20 μm

The SEM micrographs are shown in Fig. 3. SEM micrographs of random PCL fibers (3a) show typical PCL network formation with round nanofibers. Successful alignment of PCL nanofibers is demonstrated in 3b. Random (3c) and aligned (3d) PCL:elastin nanofibers exhibit significantly smaller fiber diameter than the PCL nanofibers collected under the same conditions. In addition, incorporation of elastin into the fibers caused minimal bead defects and more ribbon-like fibers.

**Fig. 4:**
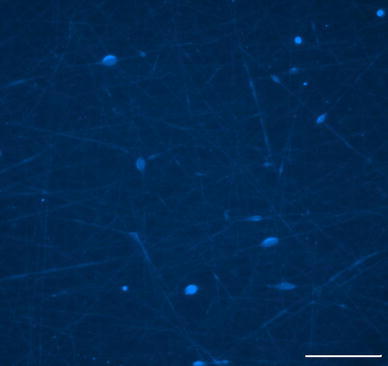
Light microscopy image of PCL scaffold with Hoechst nuclei-stained neurites. *Scale bar* is 100 μm

**Fig. 5:**
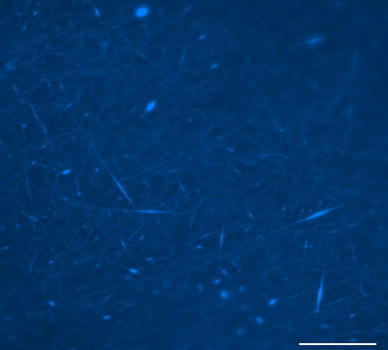
Light microscopy image of randomly oriented PCL:elastin scaffold with Hoechst nuclei-stained neurites. *Scale bar* is 100 μm

E9 chick DRG were seeded on the electrospun scaffolds to evaluate cell attachment and neurite extension. Cell attachment was significantly higher in scaffolds containing elastin than the scaffolds containing only PCL, as shown in Table 2. Cell attachment to PCL fibers after 24 h was 7.2 × 10^4^ ± 1.1 × 10^4^ cells/cm^2^ whereas cell attachment to PCL:elastin fibers averaged 1.1 × 10^5^ ± 1.1 × 10^4^ cells/cm^2^. This would be expected since elastin is an extracellular matrix protein present in the basement membrane and promotes cell adhesion. PCL has a low cell affinity due to lack of recognition sites for the cells and its hydrophobic nature (Ghasemi-Mobarakeh et al. 2008). Figure 4 shows nuclei-stained cells seeded on a PCL scaffold, while Fig. 5 shows the stained DRG on a PCL:elastin scaffold.

**Table 2:** Average cell attachment to scaffolds after 24 h ± standard error (*n* = 17)

Composition	Cells/cm^2^
PCL	7.2 × 10^4^ ± 1.1 × 10^4^
PCL:elastin	1.1 × 10^5^ ± 1.1 × 10^4^

**Fig. 6:**
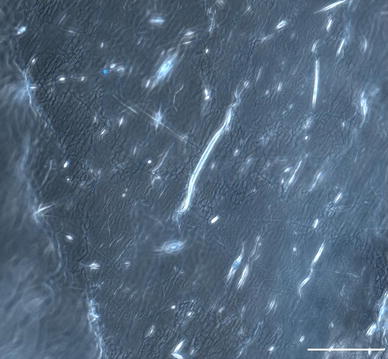
Light microscopy image of PCL:elastin aligned fibers with Hoechst nuclei-stained neurites. *Scale bar* is 100 μm

**Fig. 7:**
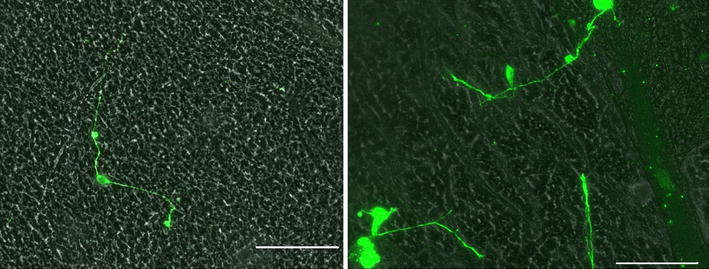
Light microscopy image of neurite extension on PCL:elastin random fibers. Neurites are stained green with beta-III-tubulin. *Scale bar* is 100 μm

**Fig. 8:**
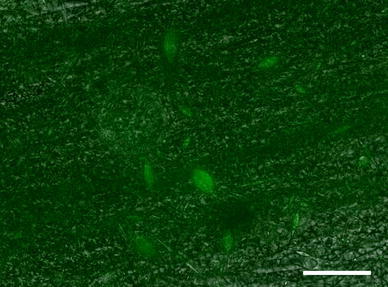
Light microscopy image of lack of neurite extension on PCL fibers. Cells are stained green with beta-III-tubulin. *Scale bar* is 100 μm

Polymer composition also plays a role in neurite extension. The neurites attached and extended along the fibers in the scaffolds containing elastin. In the scaffolds with aligned nanofibers, the cells attached and elongated along the direction of the fibers. The orientation of cell growth can be seen in the light microscopy image in Fig. 6. Fluorescent labeling of the neurites highlighted extension along the fibers in the scaffolds containing elastin. Neurite extension averaged 173.4 ± 20.7 μm (*n* = 15) after 24 h in culture. While dissociated DRGs did attach to the PCL scaffolds, there was no neurite extension in the absence of elastin. The neurite extension on PCL:elastin scaffolds is shown in Fig. 7 and lack of neurite extension on PCL scaffolds can be seen in Fig. 8.

Electrospinning is a versatile method for producing nanofibers of polymers or blends of polymers and biological components. Manipulation of the voltage, collection distance, flow rate, and solution viscosity impacts the resulting fiber diameter. The conditions were optimized for our purposes to produce nanofibers of diameters in the range of 400–1,000 nm. Fibers with diameters smaller than the size of the cell have been shown to produce faster neurite outgrowth (Corey et al. 2007). This may be due to the nanoscale fibers mimicking extracellular matrices present in the neurolemma during peripheral nerve regeneration.

Manipulation of the electric field has been used to create interesting scaffold geometries (Wang et al. 2009). In this study, the electric field was manipulated by attaching two stainless steel bolts to the collection plate. Fibers aligned themselves between the two bolts and radially from the bolts to the collection plate. This method creates aligned nanofibers without the use of a rotating mandrel, which promotes an increase in scaffold porosity.

In this study, 15 % PCL or a 4:1 blend of PCL:elastin was used to produce electrospun nanofibers. SEM micrographs revealed that the average diameter of the elastin-containing fibers was significantly less than that of the PCL fibers. This is consistent with the findings of Ghasemi-Mobarakeh et al. (2008), in which blending gelatin with PCL decreased the fiber diameter. This is due to the reduction of the solution viscosity by reducing the polymer content and increasing the elastin content. Lower solution viscosity is known to result in smaller fiber diameters. There was no significant difference between the fiber diameters of the random versus aligned scaffolds. The SEM micrographs also revealed minimal bead defects on the elastin-containing scaffolds due to the lower viscosity.

PCL has been used by many investigators as a standard polymer for electrospinning. However, it has previously been shown that additives were necessary to grow fibroblasts on a PCL scaffold (Schnell et al. 2007). This low cell affinity of PCL is in part due to lack of recognition sites for the cells and its hydrophobic nature (Ghasemi-Mobarakeh et al. 2008). In this study, E9 chick DRG were seeded on the electrospun scaffolds to evaluate cell attachment and neurite extension. More cells attached to the elastin-containing scaffolds than those containing PCL alone. This was expected because elastin has been shown to promote cell adhesion due to its presence in the basement membrane.

In addition to preferable cell attachment on the elastin-containing scaffolds, the cells appeared more elongated on the ECM-containing scaffolds. While the DRGs did attach to the PCL fibers, the incorporation of elastin promoted cell elongation along the nanofibers. This could be due in part to the observation by Wen and Tresco (2006) that neurite outgrowth decreased as the fiber diameter increased. Furthermore, cell elongation was noted in the direction of the aligned nanofibers. This observation is consistent with reports in the literature (Corey et al. 2007).

Neurite extension was quantified on the nanofiber scaffolds. While no neurite extension was seen on the PCL scaffolds, the incorporation of elastin promoted neurite extension after 24 h. While many other studies have evaluated electrospun scaffolds for neural tissue engineering, only a few have reported measurements of neurite extension (Corey et al. 2007; Schnell et al. 2007; Yang et al. 2005). Corey et al. (2007) found that after 3 days in culture, DRGs extended neurites with lengths of 760 ± 71 μm on aligned scaffolds and 631 ± 32 μm on random scaffolds. Schnell et al. (2007) saw neurite extension after 24 h of 690 ± 20 μm on PCL scaffolds and 390 ± 10 μm on PCL:collagen scaffolds. However, their sample size was small (*n* = 3), so it would be hard to draw definitive conclusions from the lengths they observed. Yang et al. (2005) observed neurite extension of 100 μm on aligned scaffolds, and 75–80 μm on random scaffolds after 24 h. Another group found that the incorporation of gelatin into the PCL scaffold promoted neurite extension (Ghasemi-Mobarakeh et al. 2008). Most of the previous studies reported qualitative results that the incorporation of a biological additive increased neurite outgrowth. Similarly, we found preferable cell attachment on the scaffolds containing elastin and neurite extension only on the elastin-containing scaffolds.

## Conclusions

Electrospun scaffolds with fiber diameters between 416 nm and 1.03 μm were repeatedly produced with minimal bead defects at 15 kV and a collection distance of 16 cm using either PCL or a 4:1 blend of PCL and elastin. Preliminary cell culture results showed the chick DRG extended neurites that attached and aligned along the polymeric nanofibers, with enhanced attachment and elongation along the scaffolds containing elastin. In addition, the cells grew in the direction of the aligned nanofibers. Future studies will incorporate elastin into the electrospun tissue engineering scaffolds due to enhanced cell attachment and elongation, and aligned fibers will be produced to direct neurite growth between the severed nerve ends.
